# Selection and Warning of Evidence-Based Antidiabetic Medications for Patients With Chronic Liver Disease

**DOI:** 10.3389/fmed.2022.839456

**Published:** 2022-02-16

**Authors:** Fu-Shun Yen, Chih-Cheng Hsu, James Cheng-Chung Wei, Ming-Chih Hou, Chii-Min Hwu

**Affiliations:** ^1^Dr. Yen's Clinic, Taoyuan, Taiwan; ^2^Institute of Population Health Sciences, National Health Research Institute, Miaoli, Taiwan; ^3^Department of Health Services Administration, China Medical University, Taichung, Taiwan; ^4^Department of Family Medicine, Min-Sheng General Hospital, Taoyuan, Taiwan; ^5^Institute of Medicine, Chung Shan Medical University, Taichung, Taiwan; ^6^Department of Medicine, Chung Shan Medical University Hospital, Taichung, Taiwan; ^7^Graduate Institute of Integrated Medicine, China Medical University, Taichung, Taiwan; ^8^Institute of Clinical Medicine, School of Medicine, National Yang-Ming Chiao Tung University, Taipei, Taiwan; ^9^Division of Gastroenterology and Hepatology, Department of Medicine, Taipei Veterans General Hospital, Taipei, Taiwan; ^10^Section of Endocrinology and Metabolism, Department of Medicine, Taipei Veterans General Hospital, Taipei, Taiwan

**Keywords:** diabetes mellitus, chronic liver disease, antidiabetic medication, compensated liver cirrhosis, decompensated liver cirrhosis

## Abstract

The global prevalence of chronic liver disease and diabetes mellitus (DM) has gradually increased potentially due to changes in diet and lifestyle. The choice of antidiabetic medications for patients with coexisting DM and chronic liver disease is complicated. Severe liver injury may decrease the metabolism of antidiabetic medications, resulting in elevated drug concentrations and adverse effects. The choice of antidiabetic medications in patients with chronic liver disease has not been well studied. The long-term outcomes of antidiabetic medications in patients with chronic liver disease have gained attention recently. Herein, we reviewed relevant articles to extend our understanding on the selection and warning of antidiabetic medications for patients with chronic liver disease.

## Introduction

The global prevalence and mortality of chronic liver disease and diabetes mellitus (DM) has gradually increased conceivably due to changes in diet and lifestyle. According to the Institute for Health Metrics and Evaluation, ~1,690 million patients had chronic liver disease including cirrhosis in 2019 (prevalence rate 22.7%), and about 1.47 million people globally died of chronic liver disease (2.6% of global all-cause mortality) (1). Moreover, according to the ninth edition of the International Diabetes Federation Diabetes Atlas in 2019, among the global adult population in the age range of 20–79 years, ~463 million patients had DM (prevalence rate 9.3%), and ~4.2 million people have died of DM (11.3% of global all-cause mortality) (2).

Chronic liver disease has long been known to be closely related to DM (3). Non-alcoholic fatty liver disease (NAFLD) is characterized by excessive triglyceride and fatty acid accumulation of liver (fat accounts for more than 5% of the liver's weight) not caused by alcohol, excessive oxidative stress, and defective insulin signaling (3). Non-alcoholic steatohepatitis (NASH) is a form of NAFLD with inflammation and damage of the liver, which can lead to hepatic fibrosis, scarring, or even cirrhosis (3, 4). Cirrhosis is characterized by diffuse nodular regeneration surrounding by a dense fibrotic septum, accompanied by the loss of liver parenchyma with collapse of the liver structure, and caused significant distortion of the hepatic vascular structure. It is the ultimate stage of chronic liver disease (5). Clinically, cirrhosis can be divided into compensated and decompensated cirrhosis (6). Patients with decompensated cirrhosis may have variceal hemorrhage, ascites, hepatic encephalopathy, jaundice, or hepatorenal syndrome. Cirrhotic patients without these complications are defined as having compensated cirrhosis (6). Patients with liver cirrhosis usually have portosystemic and intrahepatic shunt with resulting peripheral hyperinsulinemia, which can downregulate the number of insulin receptors in muscle tissues and result in insulin resistance (3). Patients with cirrhosis may be associated with beta cell dysfunction and decreased insulin secretion (5). Therefore, 60–80% patients with liver cirrhosis have glucose intolerance and 10–30% have overt type 2 diabetes (T2D) (3, 5, 7). The core protein of chronic hepatitis C virus (HCV) may impair insulin receptor substrate 1 (IRS-1) signaling and lead to insulin resistance (3). Similarly, approximately 50–70% of patients with T2D have NAFLD (3, 8, 9). T2D can accelerate the progression of chronic liver disease to liver cirrhosis and cirrhosis to subacute bacterial peritonitis, hepatic encephalopathy, and death (3, 8, 9). People with T2D also have about four times higher risk of hepatocellular carcinoma (HCC) than those without T2D (3, 5, 8).

For patients with coexisting T2D and liver cirrhosis, the choice of antidiabetic medication is complicated (3, 10). As liver is the primary site of drug metabolism, most antidiabetic medications will be metabolized in the liver and then be released to the systemic circulation. Patients with liver cirrhosis may have widespread extinction and collapse of hepatic parenchyma, which can decrease the metabolism of antidiabetic medications (9, 11). Moreover, the portosystemic and intrahepatic shunts of cirrhosis may prevent the anti-diabetic medications from entering the liver and directly go to the systemic circulation (10). Both these aforementioned phenomena may increase the systemic concentration of antidiabetic medications, leading to adverse effects.

The use of antidiabetic medications in patients with chronic liver disease has not been extensively studied (3–5, 7, 8, 12, 13). Recently, the long-term outcomes of using antidiabetic medications in patients with chronic liver disease have been uncovered. Herein, we reviewed English articles in PubMed for human studies with the following medical terms used to search: diabetes mellitus, fatty liver, steatohepatitis, liver cirrhosis, hepatocellular carcinoma; metformin, sulfonylurea, meglitinide, glinide, thiazolidinediones, rosiglitazone, pioglitazone, alpha-glucosidase inhibitor, acarbose, miglitol, voglibose, dipeptidyl peptidase-4 inhibitor, sitagliptin, vildagliptin, saxagliptin, alogliptin, linagliptin, glucagon-like peptide-1 receptor agonist, exenatide, liraglutide, lixisenatide, albiglutide, dulaglutide, semaglutide, sodium glucose cotransporters type 2 inhibitor, dapagliflozin, canagliflozin, empagliflozin, ertugliflozin, ipragliflozin, insulin; and from the references of relevant papers to summarize available evidence on the selection and warning of antidiabetic medications for patients with chronic liver disease (Table 2). Because antidiabetic medication use is complicated in patients with liver cirrhosis, the related studies are relatively few. Therefore, we present in Table 1 the literatures and results on the use of antidiabetic medications in patients with T2D and cirrhosis.

**Table 1:** Studies evaluating the impact of antidiabetic medications in patients with type 2 diabetes and liver cirrhosis.

**References**	**Study design**	**Number of patients and treatments**	**Main findings**
Nkontchou et al. (14)	Prospective	100 patients with T2D and HCV cirrhosis (metformin vs. non-metformin)	Metformin use was associated with lower risks of HCC and liver-related death or transplantation.
Ampuero et al. (15)	Retrospective cohort	82 patients with T2D and liver cirrhosis (metformin vs. non-metformin)	Metformin use seemed to be protective against hepatic encephalopathy.
Zhang et al. (16)	Retrospective	250 hospitalized patients with T2D and liver cirrhosis (continuous vs. discontinuous use of metformin)	Continuous use of metformin was associated with longer survival than those with discontinuous use of metformin.
Vilar-Gomez et al. (17)	Retrospective	191 patients with T2D and biopsy-proven NASH and fibrosis or compensated cirrhosis (metformin vs. non-metformin)	Long-term metformin use might reduce the risks of all-cause mortality or liver transplant.
Yen et al. (18)	Retrospective cohort	26,164 patients with T2D and compensated liver cirrhosis and 15,056 patients with T2D and decompensated liver cirrhosis (matched metformin users vs. non-users)	Metformin in compensated or decompensated liver cirrhosis was not associated with higher risk of metabolic acidosis. Metformin in compensated cirrhosis was associated with higher risks of mortality and cirrhotic decompensation; metformin in decompensated cirrhosis was associated with higher risk of mortality.
Yen et al. (19)	Retrospective cohort	12,078 patients with T2D and compensated liver cirrhosis (matched metformin users vs. non-users)	Sulfonylurea use was associated with significantly lower risks of mortality, major cardiovascular events, and cirrhotic decompensation.
Zillikens et al. (20)	Prospective	10 patients with alcoholic cirrhosis (acarbose vs. placebo)	Acarbose with a meal was capable of reducing blood glucose levels following that meal.
Kihara et al. (21)	Prospective	20 patients with T2D and chronic hepatitis or liver cirrhosis (acarbose vs. placebo)	Fasting plasma glucose and HbAlc levels were significantly decreased after 8 weeks of acarbose treatment.
Gentile et al. (22)	Double-blind randomized trial	100 patients with T2D and non-alcoholic liver cirrhosis (acarbose vs. placebo)	A significant reduction in fasting and postprandial glucose levels was observed after acarbose treatment. Acarbose increased bowel peristalsis, stimulated the proliferation of saccharolytic bacteria, and reduced the proliferation of proteolytic bacteria, thus reducing blood ammonia levels.
Gentile et al. (23)	Cross-over randomized trial	107 patients cirrhotic patients with grade 1-2 hepatic encephalopathy and T2D (acarbose vs. placebo)	Acarbose lowered fasting and postprandial glucose and HbA1c levels, decreased blood ammonia levels, and improved intellectual function score.
Yen et al. (24)	Retrospective cohort	10,190 patients with T2D (excluding patients with HBV infection, HCV infection, or alcoholic disorders; matched thiazolidinedione users vs. non-users)	TZD use was associated with significantly lower risk of liver cirrhosis.
Yen et al. (25)	Retrospective cohort	3,410 patients with T2D and compensated liver cirrhosis (matched thiazolidinedione users vs. non-users)	Compared with non-users, TZD users had no significant different risks of mortality, HCC, cirrhotic decompensation, and hepatic failure, but had significantly higher risk of major adverse cardiovascular events.
Yen et al. (26)	Retrospective cohort	5,656 patients with T2D and compensated liver cirrhosis (matched DPP-4 inhibitor users vs. non-users)	DPP-4 inhibitor use was not significantly associated with higher risks of mortality, cardiovascular events, and HCC but was significantly associated with higher risks of cirrhotic decompensation and hepatic failure than non-use.
Gundling et al. (12)	Retrospective	87 patients with T2D and liver cirrhosis (66% of them were on insulin therapy)	Hypoglycemia occurred especially in those with insulin therapy
Elkrief et al. (5)	Retrospective cohort	348 patients with hepatitis C-related cirrhosis (139 patients with T2D and 62% patients with diabetes were on insulin therapy)	Diabetes was independently associated with development of ascites, renal dysfunction, bacterial infections, and HCC.
Gentile et al. (27)	Double blind randomized trial	100 patients with diet-unresponsive T2D and compensated non-alcoholic liver cirrhosis (lispro vs. human regular insulin)	Lispro caused lower postprandial glucose levels and hypoglycemic risk.
Yen et al. (28)	Retrospective cohort	Patients with T2D and compensated liver cirrhosis (2,047 insulin users and 4,094 matched non-users)	Insulin use was associated with higher risks of mortality, HCC, cirrhotic decompensation, hepatic failure, cardiovascular events, and hypoglycemia than non-use of insulin.

## Management of Patients With T2D and Chronic Liver Diseases

### Diet and Lifestyle Changes

For patients with NAFLD or insulin resistance, a low-calorie, low-fat diet and increased physical activity is recommended to avoid obesity. However, for patients with liver cirrhosis, the dietary and physical recommendations are not enforced if their appetite or physical heath is not good (3, 5, 9). For those with decompensated liver cirrhosis, adequate nutrition or high-protein diet are recommended to avoid loss of muscle mass and to reduce the occurrence of ascites or edema (29). One randomized controlled trial provided a nocturnal nutritional supplement to 103 cirrhotic patients for 12 months, which resulted in protein accretion for about 2–2.5 kg of lean muscle (30). For patients with chronic liver diseases, smoking and alcohol drinking are deleterious, as they both will accelerate hepatic inflammation and increase the risks of liver cirrhosis (31, 32) and HCC (33, 34).

### Metformin

After systemic absorption, metformin enters hepatocytes through organic cation transporter (OCT), reversibly binds and inhibits the complex I of hepatic mitochondrial electron transport chain, and increase in adenosine monophosphate (AMP) production with a concomitant decrease in adenosine triphosphate (ATP) production. This activates AMP kinase (AMPK), facilitates liver kinase B1 (LKB1) phosphorylation, and negatively regulates the mammalian target of rapamycin (mTOR) pathway (35). The activation of AMPK by metformin can block hepatic glucose release and promote glucose uptake in skeletal muscles to restore insulin sensitivity and limit lipid storage in hepatocytes (35). Four randomized studies have demonstrated that metformin can improve hepatic steatosis and even fibrosis in patients with NAFLD (35). However, in three randomized trials, metformin treatment had little effect on the histological improvement of the liver (35). Three randomized and open-label studies of overweight or obese children with NAFLD found that metformin improves serum transaminase levels but not in patients with hepatic steatosis (36). Metformin may be an option for patients with NAFLD but is not recommended for those with non-alcoholic steatohepatitis (NASH) (36).

Metformin can suppress the mTOR pathway, inhibit cell proliferation, and induce apoptosis. Observational studies have suggested that metformin acts as a chemopreventive agent against HCC in patients with T2D (37). A recent meta-analysis of 19 clinical studies, including 550,882 patients with T2D and chronic liver disease, disclosed that metformin reduces the risk of HCC by 48% when administered to patients with T2D (38). However, a pooled *post-hoc* analysis of randomized control trials revealed no significant chemopreventive effect of metformin vs. placebo [adjusted odds ratio (aOR) 1.01 (0.05–21.82)] (39). One phase III study with 408 hepatitis C cirrhotic patients, compared metformin treatment with placebo for 36 months, and the primary outcome of this study was HCC occurrence and liver-related death or transplantation; unfortunately, the study was terminated by the investigator after 11 participants were accrued (40).

Metformin does not undergo hepatic metabolism and is excreted unchanged by tubular secretion and glomerular filtration into the urine (4). Although it is not expected to cause or exacerbate liver injury, physicians are often concerned about lactic acidosis when using metformin in patients with liver cirrhosis because liver cirrhosis easily leads to hepatic hypoxia, which increases anaerobic respiration and lactic acid accumulation (41). Metformin use also directly increases the production of lactic acid. However, the incidence of lactic acidosis in persons using metformin is very rare, approximately 3–10 per 100,000 person-years (42). A recent observational study disclosed that there is no unsafe plasma lactate concentration in patients with chronic liver disease and using metformin (43). In our previous study, the use of metformin in patients with compensated [adjusted hazard ratio (aHR) 1.01 (0.72–1.42)] or decompensated [aHR 0.94 (0.60–1.46)] liver cirrhosis was not associated with a higher risk of metabolic acidosis than non-use of metformin (28).

Metformin decreases portal pressure, liver injury, and improves hepatic fibrosis in cirrhotic rats (44, 45). Nkontchou et al. used a hospital-based cohort study for patients with HCV-infected liver cirrhosis, including 26 treated with metformin and 74 treated with other antidiabetic medications. This study indicated that metformin users had lower risks of HCC [aHR 0.19 (0.04–0.79)] and liver-related death or transplantation [aHR 0.22 (0.05–0.99)] than metformin nonusers (Table 1) (14). In the study by Ampuero et al. (15) on 82 cirrhotic patients with T2D, metformin use seemed to protect against hepatic encephalopathy compared with non-use of metformin [aHR of metformin non-use vs. use: 11.4 (1.2–108.8)]. Zhang et al. (16) studied a hospitalized cohort to compare 172 patients who continued metformin use for at least 3 months with 78 patients who discontinued metformin use within 3 months after the diagnosis of cirrhosis and concluded that continuous use of metformin is associated with longer survival [aHR 0.43 (0.24–0.78)]. Vilar-Gomez et al. (17)] investigated 191 patients with diabetes and biopsy-proven NASH and fibrosis or compensated cirrhosis and demonstrated that long-term metformin use reduces the risk of all-cause mortality or liver transplantation [aHR 0.42 (0.24–0.74)] and HCC [aHR 0.25 (0.11–0.58)]. We conducted a nationwide cohort study on 26,164 patients with T2D and compensated liver cirrhosis and 15,056 patients with T2D and decompensated liver cirrhosis (28). After propensity score matching, we found that metformin use in patients with compensated cirrhosis is associated with higher risks of mortality [aHR 1.13 (1.01–1.25)] and cirrhotic decompensation [aHR 1.15 (1.04–1.27)] than metformin non-use, and these higher risks were dose dependent. Metformin use in patients with decompensated cirrhosis was also significantly associated with a higher risk of mortality [aHR1.15 (1.02–1.31)] than non-use of metformin. Our contradicting results may be due to the following reasons: (1) all our patients had liver cirrhosis, with most of them having HBV or HCV infection, whereas most patients in the previous studies had NASH (16), alcoholic cirrhosis (15), HCV infected cirrhosis (14), and non-alcoholic steatohepatitis, fibrosis, or cirrhosis (17); (2) all five studies were conducted on patients with different ethnicities; (3) the patient numbers and study designs differed greatly.

**Figure 1:**
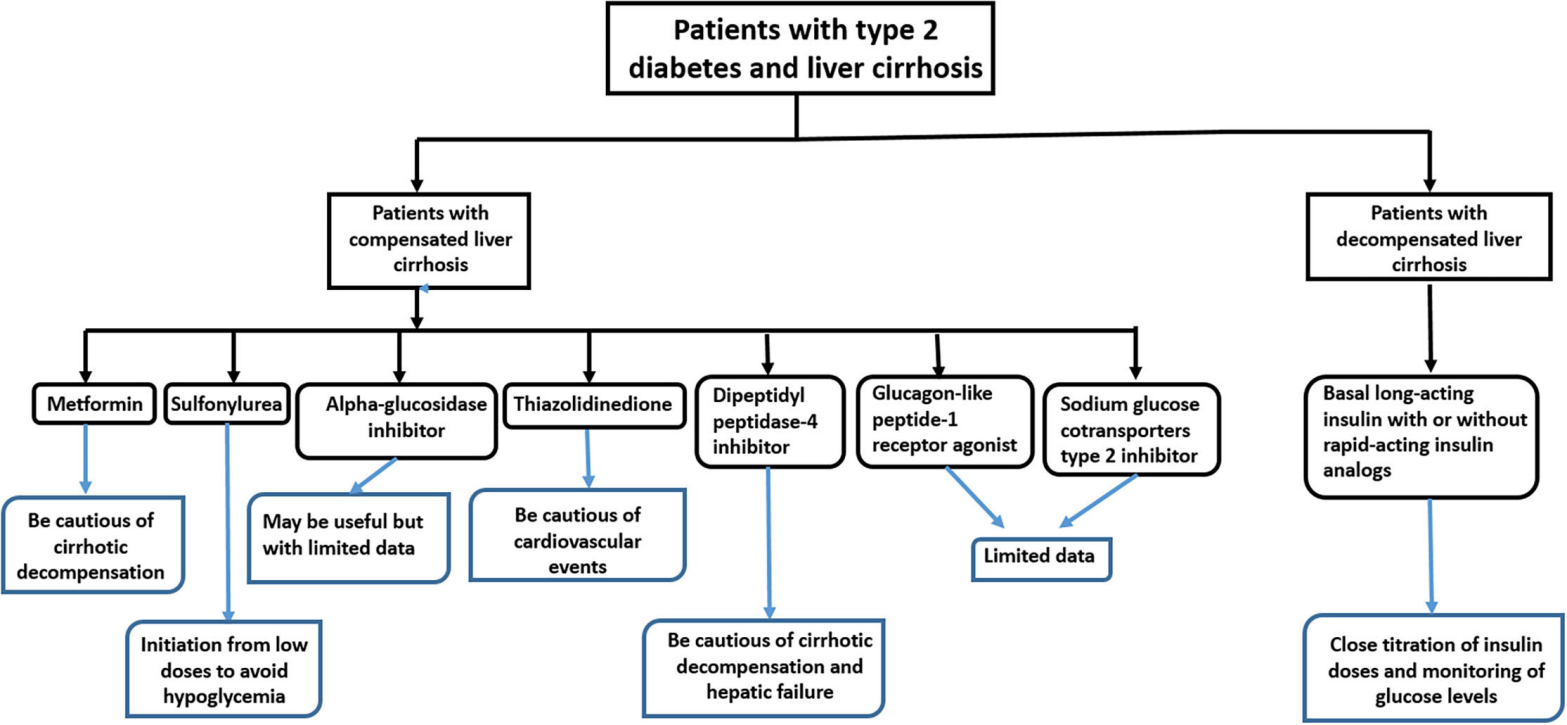
The selection and warning of antidiabetic medications for patients with type 2 diabetes and liver cirrhosis.

In brief, metformin may be useful for patients with NAFLD, but its benefit for patients with NASH is inconclusive (Table 2). It may be useful for preventing HCC occurrence, but randomized controlled trials have not confirmed this. It may be useful for patients with compensated liver cirrhosis, but our study indicated higher risks of cirrhotic decompensation in metformin users (Figure 1). It may not be recommended for patients with decompensated cirrhosis (Table 2).

**Table 2:** The usefulness and warning of antidiabetic medications in patients with chronic liver diseases.

**Medication**	**Mechanism of action**	**Fatty liver diseases**	**Compensated liver cirrhosis**	**Decompensated liver cirrhosis**
Metformin	Restore insulin sensitivity	Useful for NAFLD, but may not for NASH	May be useful, but may need to be cautious of mortality and decompensated cirrhosis.	Limited data, but may need to be cautious of mortality.
Sulfonylurea	Increase insulin secretion	Not useful	Useful, but initiation from low doses to avoid hypoglycemia	Not recommended
Meglitinide	Acutely increase insulin secretion	Limited data	Limited data	Not recommended
Thiazolidinedione	Increase systemic insulin sensitivity	Useful for NAFLD and NASH	May be useful, but may need to be cautious of major cardiovascular events	Limited data
Alpha-glucosidase inhibitor	Delay intestinal carbohydrate absorption	Not useful	May be useful; acarbose may decrease ammonia levels and improve intellectual function in patients with low-grade hepatic encephalopathy	Not recommended
DPP-4 inhibitor	Prolong the activity of GLP-1 and GIP and stimulate insulin secretion	Useful for NAFLD	May be useful, but may need to be cautious of cirrhotic decompensation and hepatic failure	Limited data
GLP-1 receptor agonist	Glucose-dependent stimulate insulin secretion and inhibit glucagon release	Useful for NAFLD and NASH	Limited data	Limited data
SGLT2 inhibitor	Promote urinary glucose excretion	Useful for NAFLD	Limited data	Not recommended
Insulin	Substitutive treatment	Not useful	May not be useful, need to be cautious of mortality, HCC, cirrhotic decompensation, hepatic failure, cardiovascular events, and hypoglycemia	May be useful to use basal long-acting insulin analogs with or without rapid-acting insulin analogs, combined with close titration of insulin doses and monitoring of glucose levels

### Sulfonylureas (SUs)

SUs bind to the SU receptor on pancreatic beta cells, resulting in the closure of potassium channels, inhibition of potassium efflux, and increased influx of calcium. The influx of calcium causes microtubule contraction and exocytosis of insulin from secretory vesicles (46). The main adverse effect of SUs is hypoglycemia. The average incidence of mild or moderate hypoglycemia is 1–2% per year, but prolonged and severe hypoglycemia can occur in patients with severe renal or hepatic impairment (47). SUs are predominantly metabolized by the liver and cleared by the kidneys (3). Their hepatic metabolism may decrease in patients with chronic liver disease, and the systemic concentration may increase. It is recommended to closely monitor the blood glucose levels during SU treatment in patients with advanced liver disease to avoid hypoglycemia.

Sulfonylureas can induce pancreatic beta cells to secrete insulin, a growth-promoting hormone with mitogenic effects. One meta-analysis of eight observational studies disclosed that SU use was associated with a 62% higher risk [aOR 1.62 (1.16–2.24)] of HCC in patients with T2D than non-use of SU (39). In 3,781 selected matched pairs of SU users and non-users among patients with T2D and compensated liver cirrhosis, the risk of HCC [aHR 0.99 (0.90–1.11)] was not significantly different between SU users and non-users (26).

The UK Prospective Diabetes Study (UKPDS) demonstrated that intensive glycemic control with SUs or insulin significantly reduces macrovascular complications associated with improved glycemic control (48). SU-induced hepatotoxicity has rarely been reported for glycemic control in patients with T2D (11). Glibenclamide significantly increased portal and systemic vascular resistance initially, then decreased portal pressure and increase systemic vascular resistance in cirrhotic rats (49). However, few studies have investigated the use of SUs in patients with liver cirrhosis. Singh et al. (50) used tolbutamide to treat 55 patients with liver cirrhosis; in 35 patients, the protein level in the serum returned to normal, and 42 of them were relieved of ascites. We conducted a retrospective cohort study to investigate the long-term outcomes of SU use in patients with T2D and compensated liver cirrhosis (24). After propensity score matching, 3,781 pairs of SU users and non-users were selected; SU users had significantly lower risks of all-cause mortality [aHR 0.79 (0.71–0.88)], major cardiovascular events [including stroke, ischemic heart disease, and heart failure, aHR 0.69 (0.61–0.80)], and decompensated cirrhosis [including variceal bleeding, ascites, hepatic encephalopathy, and jaundice; aHR 0.82 (0.66–1.03)] (18). The lower risks of death, cardiovascular events, and hepatic outcomes associated with SU use in this study may be attributed to the glucose-lowering effect of SUs, as indicated in the UKPDS and other studies on the glucose-lowering effects of SUs (48).

SUs may be useful for patients with compensated liver cirrhosis (Table 2); however, they must be initiated at low doses to avoid hypoglycemia. Moreover, due to limited data, they are not recommended for patients with decompensated liver cirrhosis (4).

### Meglitinides (Glinides)

Meglitinides (including repaglinide, nateglinide, and mitiglinide) bind to the SU receptor at a different site than the SUs. Their onset of action is faster, and the half-life is shorter, which results in a brief stimulation of insulin release (46). Because they briefly stimulate insulin secretion, which can reduce the risk of hypoglycemia, meglitinides are especially good for people with hepatic or renal impairment and elderly patients (11). Their risk of hypoglycemia is lower than that of SUs because of their shorter duration of action and glucose-dependent insulinotropic effects (46). These compounds are metabolized and secreted by the liver to inactive biliary products. The pharmacokinetics and tolerability of nateglinide in patients with compensated cirrhosis are not significantly different from that in healthy individuals (51). However, the clearance of repaglinide is significantly lower in patients with chronic liver disease (nine patients were Child-Pugh B and three were Child-Pugh C) than in healthy individuals; therefore, it should be used with caution in patients with liver cirrhosis (52). One randomized study compared five patients with T2D and NASH using nateglinide with five patients not using nateglinide; nateglinide-treated patients had improved liver function and histological findings (53). However, the long-term outcome of meglitinide use in patients with chronic liver disease has not been reported.

Meglitinides should be initiated at low doses in patients with compensated liver cirrhosis to avoid hypoglycemia and are not recommended for patients with decompensated liver cirrhosis (4).

### Alpha-Glucosidase Inhibitors (AGIs)

AGIs including acarbose, miglitol, and voglibose competitively inhibit alpha-glucosidase in the brush border of the small intestine (46). Alpha-glucosidase breaks down oligosaccharides and disaccharides into monosaccharides. Therefore, its inhibition by AGIs will delay the absorption of carbohydrates, resulting in lower postprandial glucose levels (46). Acarbose, which is not absorbed intestinally and does not undergo hepatic metabolism, was documented to have good tolerability with no toxic effects on the liver (11). Because acarbose reduces body mass index, waist circumference, and triglyceride levels, it may be a promising antidiabetic drug for the treatment of patients with NASH (54). It can be safely used in patients with T2D and chronic liver disease (21), compensated non-alcoholic cirrhosis (22), and alcoholic liver cirrhosis (20) and can prevent fasting and postprandial hyperglycemia (Table 1). In a crossover randomized study including patients with T2D and grade 1–2 hepatic encephalopathy, acarbose significantly decreased blood ammonia levels and improved intellectual function score and postprandial glucose levels compared with placebo (23). Acarbose increases bowel frequency, induces the proliferation of saccharolytic bacteria, and inhibits the proliferation of proteolytic bacteria, all of which reduce intestinal ammonia production (22).

AGIs can be safely used in patients with compensated liver cirrhosis, but they may not be recommended for patients with decompensated liver cirrhosis (Table 2).

### Thiazolidinediones (TZDs)

TZDs (including pioglitazone and rosiglitazone) are ligands for the peroxisome proliferator-activated receptor gamma (PPARγ), which regulates the expression of genes involved in carbohydrate and lipid metabolism (46). TZDs can ameliorate insulin resistance, improve glucose metabolism, stimulate fatty acid oxidation, and inhibit hepatic fatty acid synthesis. The major adverse effects of TZDs are weight gain, edema, and congestive heart failure; therefore, they are contraindicated in patients with class II-IV congestive heart failure (46). TZDs are metabolized and excreted via the liver rather than the kidneys; therefore, they should be used carefully in patients with compensated liver cirrhosis and should not be prescribed to patients with decompensated cirrhosis (11). Belfort et al. (55) randomly compared a hypocaloric diet plus pioglitazone with the diet plus placebo in 55 patients with impaired glucose tolerance or T2D. The pioglitazone group showed decreased liver function and hepatic fat content and improved histologic steatosis but no significant improvement in fibrosis compared with the placebo group. Cusi et al. (56) performed a similar randomized controlled trial to compare 101 patients with prediabetes or T2D (consuming hypocaloric diet plus pioglitazone or the diet plus placebo); the pioglitazone group showed reduced liver triglyceride content, improved histological steatosis, and fibrosis. Aithal et al. (57) conducted a randomized controlled trial to compare the effect of standard diet and exercise along with pioglitazone or along with placebo in 74 non-diabetic patients with NASH. The pioglitazone group showed reduced alanine aminotransferase levels, improved histologic features of hepatic injury, and fibrosis compared with the placebo group. Sanyal et al. (58) performed a randomized controlled trial to compare the effect of vitamin E plus pioglitazone and vitamin E plus placebo in non-diabetic patients with NASH. Treatment with vitamin E and pioglitazone reduced the aminotransferase levels and decreased hepatic steatosis but could not improve fibrosis compared with compared with treatment with vitamin E plus pioglitazone (58). Ratziu et al. (59) randomly assigned 63 patients with NASH to receive rosiglitazone or placebo treatment. The rosiglitazone group showed 21% of the patients improvement in hepatic steatosis and 21% normalization of transaminase levels, but no improvement in fibrosis was noted (59). One systematic review and meta-analysis of TZD use in patients with NASH indicated that TZD decreases hepatic fat content, normalizes aminotransferase levels, and improves histological steatosis (60). These studies indicate that TZD use in patients with NASH could attenuate hepatic injury, inflammation, and even fibrosis. We conducted a cohort study in patients with newly diagnosed T2D (excluding patients with HBV or HCV infection or alcoholic disorders) to compare the liver outcomes between 5,095 paired TZD users and non-users. TZD users had a significantly lower risk of liver cirrhosis than non-users [aHR 0.39 (0.21–0.72)] (24). We conducted another cohort study to investigate the long-term outcomes of TZD use vs. non-use in 3,410 patients with compensated liver cirrhosis. Risks of all-cause mortality, HCC, cirrhotic decompensation, and hepatic failure did not differ between TZD users and non-users, but TZD users had a significantly higher risk [aHR 1.70 (1.32–2.19)] of major adverse cardiovascular events (composite ischemic heart disease, stroke, and heart failure) than non-users (28). Our studies suggest that in patients with NAFLD or fibrosis, TZD may be able to slow disease progression (Table 2); however, in the stage of compensated cirrhosis, it may be too late to attenuate cirrhotic deterioration (Table 1) (61).

TZDs activate PPARγ to induce cell cycle arrest and apoptosis and inhibit cancer cell proliferation and invasion (62). TZD use may decrease the risk of HCC in patients with T2D (63, 64). However, one meta-analysis (39) and our studies (18, 19) did not exhibit a lower risk of HCC in TZD users.

TZDs may be useful for patients with NAFLD, NASH, and chronic liver diseases, but need to be cautious of major cardiovascular events in patients with liver cirrhosis.

### Dipeptidyl Peptidase-4 (DPP-4) Inhibitors

Blocking of DPP-4, which is required for degrading glucagon-like peptide-1 (GLP-1), and glucose-dependent insulinotropic polypeptide (GIP), DPP-4 inhibitors prolong the activity of GLP-1 and GIP. Both GLP-1 and GIP can stimulate pancreatic beta cells to secrete insulin in a glucose-dependent manner to control blood glucose levels with hypoglycemia occurring very rarely as an adverse effect (65). Most DPP-4 inhibitors are metabolized in the kidney. They have been well studied in patients with various degrees of chronic liver diseases. The pharmacokinetic and safety profiles of DPP-4 inhibitors are generally good in patients with compensated liver cirrhosis, but data on patients with decompensated liver cirrhosis are limited (11). Because DPP-4 is upregulated in patients with chronic liver disease, DPP-4 inhibitors may have good therapeutic effects in these patients (66). Sitagliptin has been reported to improve liver function in patients with T2D and NAFLD (67). Two randomized controlled trials determined the effect of 1-year sitagliptin treatment in patients with NASH and found that sitagliptin improved hepatic steatosis and NASH activity score; however, the extent of fibrosis was unchanged (68, 69). One randomized study demonstrated that 6 months of vildagliptin therapy could significantly decrease hepatic triglyceride and plasma alanine aminotransferase levels compared with the placebo (70). We conducted a retrospective cohort study to determine the long-term outcomes of DPP-4 inhibitors use in patients with T2D and compensated liver cirrhosis. Our study disclosed that DPP-4 inhibitors were not significantly associated with higher risks of all-cause mortality, major cardiovascular events, and HCC but were significantly associated with higher risks of cirrhotic decompensation [aHR 1.35 (1.03–1.77)] and hepatic failure [aHR 1.35 (1.02–1.79)] than non-users (Table 1) (28). DPP-4 inhibitors can increase GLP-1 and GIP levels in splanchnic and portal circulation, which can promote nitro oxide production, accelerate portal vein inflow, and increase portal pressure (28).

DPP-4 inhibitors may be useful in patients with NAFLD and chronic liver disease, but the patients must be monitored for cirrhotic decompensation and hepatic failure in patients with liver cirrhosis (Figure 1).

### Glucagon-Like Peptide-1 (GLP-1) Receptor Agonists

GLP-1 receptor agonists stimulate insulin secretion and inhibit glucagon release by the beta and alpha cells of the pancreas, thereby reducing postprandial glucose levels (71). They also decrease gastric emptying time and body weight. GLP-1 receptor agonists are rarely metabolized by the liver and are excreted unchanged by the kidney; therefore, they may be safely used in patients with compensated cirrhosis (11). Two studies have demonstrated that the use of GLP-1 receptor agonists in patients with NAFLD and T2D could reduce intrahepatic fat content (72, 73). The combination of liraglutide with sitagliptin or pioglitazone has been assessed in Japanese patients with NAFLD and T2D. Liraglutide improved glycemic parameters and reduced body weight, inflammation, and liver fibrosis (74). A meta-analysis of the “Liraglutide Effect and Action in Diabetes” program showed liraglutide therapy is safe and tolerated well and improves the levels of liver enzymes in patients with T2D (75). In a 72-week randomized control trial involving 320 patients with biopsy-confirmed NASH and liver fibrosis, patients were randomly assigned to receive once-daily subcutaneous semaglutide or corresponding placebo. This study demonstrated that treatment with semaglutide resulted in a significantly higher percentage of patients with NASH resolution than placebo, but fibrosis stage did not significantly improve (76). The potential mechanism of GLP-1 receptor agonists in NASH may relate to weight loss, reduced insulin resistance, and metabolic dysfunction (76).

GLP-1 receptor agonists may be useful for patients with NAFLD or NASH. Unfortunately, the effect of the long-term administration of these drugs in patients with chronic liver disease, particularly those with liver cirrhosis, is not known (Table 2).

### Sodium Glucose Cotransporters Type 2 (SGLT2) Inhibitors

SGLT2 inhibitors promote urinary glucose excretion, decrease blood glucose levels, and improve insulin resistance in patients with T2D (77). Improvement in hyperglycemia can downregulate carbohydrate-responsive element-binding protein (ChREBP) and reduce fatty acid synthesis. Improvement in insulin resistance can downregulate sterol regulatory element-binding protein 1c (SREBP-1c) and block de novo hepatic lipogenesis (78). No clinically relevant changes in pharmacokinetic parameters have been observed in patients with T2D and mild or moderate hepatic impairment, and data from a large phase II–III trial have shown that SGLT2 inhibitors do not cause hepatotoxicity (11). However, they should be used with caution and in lower doses in patients with cirrhosis to avoid the risks of dehydration and hypotension (4). Ipragliflozin (79, 80) and luseogliflozin (81) have been demonstrated to reduce liver fat in Japanese patients with T2D and NAFLD. A *post-hoc* analysis of the EMPA-REG OUTCOME® trial showed that empagliflozin could reduce liver fat and aminotransferase levels in individuals with T2D (82). In a randomized controlled trial, empagliflozin was demonstrated to reduce liver fat and improve alanine transaminase levels in patients with T2D and NAFLD, but this effect did not correlate with glycemic improvement or body weight reduction (78). Studies on patients with T2D and NAFLD showed that dapagliflozin improves liver function and hepatic fat content (83, 84).

SGLT2 inhibitors, through inhibiting the reabsorption of glucose and sodium in renal proximal tubules, can induce natriuresis and attenuate renin secretion (85). There have been case reports using SGLT2 inhibitors to ameliorate ascites and peripheral edema in patients with liver cirrhosis; one of the cases also used spironolactone, and another case was on propranolol (86). SGLT2 inhibitors have been used with loop diuretics to increase natriuresis in patients with heart failure (87). But close monitoring of SGLT2 inhibitors plus diuretics is necessary to avoid the risks of hypovolemia, hypotension, encephalopathy and hepatorenal syndrome in patients with cirrhosis (86). One study has reported that the coadministration of SGLT2 inhibitors and β blocker did not affect the eGFR response to SGLT2 inhibitors (88). Therefore, it may be safe to co-administer SGLT2 inhibitors with β blockers in patients with liver cirrhosis.

SGLT2 inhibitors may be useful for patients with NAFLD, but because they have only been introduced in the market in 2016, the long-term outcomes of their use in chronic liver disease have not yet been reported (Figure 1).

### Insulin

Insulin therapy is considered the safest and most effective antidiabetic management in patients with chronic liver disease, but it is associated with an increased risk of hypoglycemia (4, 11). The liver is the major site of metabolism for circulating insulin, and ~40–50% of the endogenous insulin is metabolized by the liver (4). Different patients with liver cirrhosis require different levels of insulin. Patients with compensated liver cirrhosis may have a higher insulin requirement because of the prominent insulin resistance. However, in patients with decompensated cirrhosis, the hepatic metabolism of insulin is reduced, thereby reducing the need for insulin (8). Therefore, therapy with insulin in patients with liver cirrhosis requires close monitoring of blood glucose levels to avoid the risks of hypoglycemia or hyperglycemia (8).

The use of insulin in patients with chronic liver disease is associated with an increased HCC risk (39, 89). However, insulin has been reported to decrease intrahepatic fat content in drug-naïve patients with T2D (90). It reversed major portal hypertension-related derangements in rats with diabetes and liver cirrhosis (91). Elkrief et al. (92) reported that 62% of 348 patients with hepatitis C-related cirrhosis were on insulin therapy (Table 1). Gundling et al. (12) reported that 66% of 87 patients with T2D and liver cirrhosis were on insulin therapy, and hypoglycemia occurred especially in those undergoing insulin therapy. Gentile et al. (27) compared the metabolic profiles of lispro and human regular insulin in patients with diet-unresponsive T2D and compensated non-alcoholic liver cirrhosis and found that lispro caused lower postprandial glucose levels and carried a lower hypoglycemia risk. We have conducted a retrospective cohort study to investigate the long-term outcomes of insulin use in persons with T2D and compensated liver cirrhosis. Our study revealed that insulin use is associated with higher risks of all-cause mortality [aHR 1.31 (1.18–1.45)], HCC [aHR 1.18 (1.05–1.34)], decompensated cirrhosis [aHR 1.53 (1.35–1.72)], hepatic failure [aHR 1.26 (1.42–1.86)], major cardiovascular events [aHR 1.41(1.23–1.62)], and hypoglycemia [aHR 3.33 (2.45–4.53)] than non-use of insulin (28). Although we cannot completely exclude the bias of cofounding by indication that physicians may choose to prescribe insulin therapy for patients with more severe cirrhosis, our study suggested that in patients with compensated liver cirrhosis, the use of insulin warranted special attention. Currently, there are no guidelines recommending the best insulin preparation to treat patients with T2D and liver cirrhosis. Because patients with decompensated liver cirrhosis usually need to be hospitalized, it may be appropriate to treat these patients following the guidelines of diabetes treatment for inpatients (93). Accordingly, they can initially be treated with long-acting insulin analogs (insulin glargine, detemir, and degludec) because they are more stable, exhibit persistent effects, and carry lower risks of glucose fluctuation and hypoglycemia compared with neutral protamine Hagedorn-insulin and premixed insulin (13). Rapid-acting insulin analogs (insulin lispro, aspart, and glulisine) may then be added as needed because they can rapidly decrease blood glucose levels while carrying a lower risk of postprandial hypoglycemia than regular short–acting insulin (27). Combined with close titration of insulin doses and monitoring of blood glucose, insulin therapy may be a safe and effective antidiabetic management strategy in patients with decompensated liver cirrhosis (Figure 1).

In brief, insulin may be useful in patients with compensated and decompensated liver cirrhosis, but close titration of insulin doses and frequent monitoring of glucose levels are needed to avoid the risk of hypoglycemia.

## Perspectives

Metformin, thiazolidinediones, DPP-4 inhibitors, GLP-1 receptor agonists, and SGLT2 inhibitors may be useful for patients with NAFLD or NASH, but only thiazolidinediones may be able to attenuate fibrosis or even cirrhosis (Table 2). Metformin may decrease the risk of HCC, but this effect needs to be confirmed through a randomized controlled trial. Metformin, SUs, meglitinides, alpha-glucosidase inhibitors, TZDs, DPP-4 inhibitors, GLP-1 receptor agonists, and SGLT2 inhibitors may be used in patients with compensated liver cirrhosis; however, patients treated with metformin and DPP-4 inhibitors must be monitored for cirrhotic decompensation, those treated with TZDs need to be monitored for cardiovascular events, and those treated with SUs and meglitinides must be initially prescribed low doses to avoid hypoglycemia. The long-term outcomes of treatment with meglitinides, alpha-glucosidase inhibitors, GLP-1 receptor agonists, and SGLT2 inhibitors in these patients have not yet been reported. Given their fragility and frequent admission, it is recommended to treat patients with decompensated liver cirrhosis with basal long-acting insulin analogs with or without rapid-acting insulin analogs, carefully titrating the insulin doses and closely monitoring the blood glucose levels (Figure 1).

## Author Contributions

F-SY, C-CH, and M-CH wrote the manuscript. JW and C-MH made critical revisions. All authors approved the final version.

## Funding

This study was supported by the grants from the Taipei Veterans General Hospital (V105C-204, V110C-175, V109C-189, V108C-172, and VN107-07).

## Conflict of Interest

The authors declare that the research was conducted in the absence of any commercial or financial relationships that could be construed as a potential conflict of interest.

## Publisher's Note

All claims expressed in this article are solely those of the authors and do not necessarily represent those of their affiliated organizations, or those of the publisher, the editors and the reviewers. Any product that may be evaluated in this article, or claim that may be made by its manufacturer, is not guaranteed or endorsed by the publisher.

## Abbreviations

DM: diabetes mellitus
T2D: type 2 diabetes
HbA1c: hemoglobin AlC
HCV: hepatitis C virus
HBV: hepatitis B virus
HCC: hepatocellular carcinoma
NAFLD: non-alcoholic fatty liver disease
NASH: non-alcoholic steatohepatitis
SU: Sulfonylurea
AGI: Alpha-glucosidase Inhibitor
TZD: Thiazolidinedione
DPP-4 inhibitors: dipeptidyl peptidase-4 inhibitors
GLP-1: glucagon-like peptide-1
SGLT2: sodium glucose cotransporters type 2.
